# Social Distancing and COVID-19: Factors Associated With Compliance With Social Distancing Norms in Spain

**DOI:** 10.3389/fpsyg.2021.727225

**Published:** 2021-09-14

**Authors:** Estrella Gualda, Andre Krouwel, Marisol Palacios-Gálvez, Elena Morales-Marente, Iván Rodríguez-Pascual, E. Begoña García-Navarro

**Affiliations:** ^1^Social Studies and Social Intervention Research Center, Contemporary Thinking and Innovation for Social Development Research Center, Faculty of Social Work, University of Huelva, Huelva, Spain; ^2^Faculty of Social Sciences, Vrije Universiteit Amsterdam, Amsterdam, Netherlands; ^3^Contemporary Thinking and Innovation for Social Development, Faculty of Education, Psychology and Sport Sciences, University of Huelva, Huelva, Spain; ^4^Social Studies and Social Intervention Research Center, Contemporary Thinking and Innovation for Social Development Research Center, Faculty of Nursing, University of Huelva, Huelva, Spain

**Keywords:** social distancing, COVID-19, coronavirus, disinformation, conspiracy theories, public health, pandemics, Spain

## Abstract

This article describes patterns of compliance with social distancing measures among the Spanish population during the coronavirus disease-2019 (COVID-19) pandemic. It identifies several factors associated with higher or lower compliance with recommended measures of social distancing. This research is part of a 67-country study, titled the *International COVID-19 study on Social & Moral Psychology*, in which we use a Spanish dataset. Participants were residents in Spain aged 18 or above. The sample comprises 1,090 respondents, weighted to be representative of the Spanish population. Frequencies, correlations, bivariate analysis, and six models based on hierarchical multiple regressions were applied. The main finding is that most Spaniards are compliant with established guidelines of social distance during the pandemic (State of Alarm, before May 2020). Variables associated more with lower levels of compliance with these standards were explored. Six hierarchical multiple regression models found that compliance with social distance measures has a multifactorial explanation (*R*^2^ between 20.4 and 49.1%). Sociodemographic factors, personal hygiene patterns, and the interaction between personal hygiene patterns and the support for political measures related to the coronavirus brought significant effects on the regression models. Less compliance was also associated with beliefs in some specific conspiracy theories with regard to COVID-19 or general conspiracy mentality (Conspiracy Mentality Questionnaire, CMQ), consumption patterns of traditional mass media (television, paper newspapers, magazines, and radio) and modern means to get informed (online digital newspapers, blogs, and social networks), political ideology, vote, trust in institutions, and political identification. Among the future lines of action in preventing the possible outbreak of the virus, we suggest measures to reinforce trust in official information, mainly linked to reducing the influence of disinformation and conspiracy theories parallel to the pandemic.

## Introduction

After the outbreak of the worldwide coronavirus disease-2019 (COVID-19) pandemic, it became clear already, in the first months after its eruption, that there were vast differences in how countries tried to curb the spread of the virus. Political and public health measures to slow the expansion of the pandemic and decisions on how to manage it varied substantially across nations. It led to spatial and temporal heterogeneity in the COVID-19 spread (Yamamoto et al., 2021), while sociocultural practices associated with the observance of social distance during the epidemic, such as religious and tribal aspects, also matter (Mansdorf, 2020; Martinez-Brawley and Gualda, 2020). Also, COVID-19 cumulative mortality over the first pandemic wave varied widely across countries, as Piovani et al. (2021) addressed. Their study documented the importance of adopting social distancing measures soon as mass gatherings restrictions and school closures. These measures contributed to reduce COVID-19 cumulative mortality during the first pandemic wave.

However, despite recommendations to adopt rapid preventive measures, identical policies of restrictions of freedom, confinement, and social distancing have not been articulated in all countries in the same manner or with the same intensity (Travaglino and Moon, 2021; Yamamoto et al., 2021). Differences in managing the pandemic were found even having a common frame of basic recommendations of the WHO regarding their effectiveness in reducing rates of contagion and mortality, along with other critical well-known proposals to prevent and confront COVID-19: hand hygiene (with soap or alcoholic gel if the former is not available), avoiding touching of the eyes, nose, and mouth, protecting others by covering mouth while coughing, cleaning surfaces, or using a mask in certain situations (among others) (World Health Organization, 2020).

On January 31, 2020, the new coronavirus was declared a global emergency by the WHO. At that moment, nobody could imagine the quick spread and high lethality, especially for vulnerable groups. More than a year after this declaration, more than 4 million deaths and nearly 190 million COVID-19 confirmed cases have been registered in the world [July 14, 2021] (https://coronavirus.jhu.edu/map.html), data that clearly show the seriousness of this pandemic (Johns Hopkins University, 2021).

Spain, along with Italy, has been one of the most affected countries in Europe by the first wave of COVID-19 and one of the places in the world with higher mortality rates for COVID-19 in the first phase of the epidemic. Moreover, a year later, Spain represented one of the places in the world with a higher number of cases and deaths reported (John Hopkins University, https://coronavirus.jhu.edu/map.html). This article is based precisely on the first wave. In the case of Spain, the government declared a “State of Alarm” [Estado de alarma] on March 14, 2020 [Royal Decree No. 463 (Real Decreto 463/2020, 2020)], which, in the context of maximum emergency, restricted freedom and applied social distance measures to reduce the curve of infections and minimize the impact of the virus on the health system. In later phases, de-escalation measures especially emphasized the need for social and individual responsibility to avoid outbreaks. Thus, in the face of lockdown measures or deprivations of freedom, such as mobility, social distance measures are less rigid but more challenging to monitor by relying on the consent of the individual to modify routines.

Not all citizens react in the same way to restrictions, although the success of public health measures for controlling the coronavirus pandemic relies on population compliance to health measures (Sparrow et al., 2020; Zhao et al., 2020). Recent literature has reported several risk factors to explain the achievement of compliance of social distancing norms or protective behaviors during the pandemic (Mansdorf, 2020; Trifiletti et al., 2021). Aspects as being highly stressed (Constantinou et al., 2021), the connection between intentions and behaviors (Smith and Branscum, 2021), mental health symptoms or mental disorders (Stein et al., 2020; Zhao et al., 2020), public trust or trust in government (Pak et al., 2021; Travaglino and Moon, 2021), or perceived social support (Toktam et al., 2020) have been assessed in recent bibliography in connection to compliance with social distancing norms. Likewise, sociodemographic, economic, and political factors such as sex, age, race, marital family status, having children, income, work, ideology, and vote (Margraf et al., 2020; Woelfert and Kunst, 2020; Papageorge et al., 2021; Smith and Branscum, 2021; Uddin et al., 2021) have been considered in the literature to explore observance behaviors and compliance with social distancing during the pandemic.

In Spain, several contagion curve peaks during the COVID-19 pandemic were explained, among other factors, by relaxation of the behavior of its citizens (Albalá, 2020; Casares and Khan, 2020; Infosalus, 2020; Agencia Efe, 2021; Cuello-Díaz, 2021). News in Spain during the pandemic has been continuously informing of the connection between infections and social behavior (Albalá, 2020). Moreover, even the Spanish government referred to the relaxation of behaviors or established strategies to combat it, for instance, recommending doing a communication campaign to avoid relaxation of behaviors, a campaign to reduce the impact of ≪pandemic fatigue≫ during festivities such as San José or the Holy Week festivity this year (Ministerio de Sanidad, 2021).

At the beginning of the pandemic, strict confinement measures for the first phase were highly influential in containing the development of the virus in Spain. Nevertheless, once the state of alarm had ended, government communications linked some peaks of contagion with celebrations, parties, or the relaxation of social distance in environments such as family or social. For example, the Center for the Coordination of Health Alerts and Emergencies (Centro de Coordinación de Alertas y Emergencias Sanitarias) of the Ministry of Health explained in its May 21, 2021 report, concerning cases that have been traced and associated with an outbreak, that “Family meetings (non-cohabitants) and friends continue to be the area of most frequent occurrence, followed by catering establishments (restaurants, bars, cafes…) and sports activities” (Centro de Coordinación de Alertas y Emergencias Sanitarias, 2021, own translation).

Compliance with pandemic control measures, such as social distancing, is primarily the responsibility of citizens themselves, and its effectiveness was considered vital by many governments for the reduction of rate of spread and containment of the virus (Briscese et al., 2020; Painter and Qui, 2021). It has been anticipated that compliance with social distancing norms would rely on more informal social interactional processes, suggesting tensions and polarization between those who support and do not support social distancing (“distancers” and “non-distancers”) (Prosser et al., 2020). Polarization would happen, especially after the stricter lockdown phase.

Some authors remarked that the practice of social distancing and mask-wearing has been controversial and even politicized (Xu and Cheng, 2021). Others emphasized that many citizens did not comply fully with the measures (Murphy et al., 2020; Sparrow et al., 2020). Also, there have frequently been other reports referring to parts of the population that do not rigorously follow social distancing measures, putting public health at risk and overloading health services.

These difficulties in following public health guidelines are an old and well-known problem in medicine (Mansdorf, 2020). Thus, if people usually do not do what is right and refuse to stop harmful behaviors to them and others, it is expected that the same behavior will occur with regard to the restrictions derived from COVID-19. In his opinion, what makes the difference in the case of the airborne infectious COVID-19 is that this type of behavior of non-observance of social distancing measures puts other citizens at risk and poses real threats to national health, security, and economy. It makes a difference compared with, for example, the fact that a person consumes many sugary beverages or does not take medication.

On the other side, part of the citizenry mistrusts official information regarding COVID-19, while large doses of disinformation and conspiracy theories are deployed. International authorities have highlighted these aspects as an obstacle to the management of the pandemic. WHO Director-General Dr. Tedros Adhanom, declared on February 15, 2020, “we were fighting against both an epidemic and an infodemic”, and in his words: “But we're not just fighting an epidemic; we're fighting an infodemic. Fake news spreads faster and more easily than this virus and is just as dangerous” (Adhanom, 2020, para. 45–46). This peculiarity requires working to counter the infodemic, which is as dangerous as the virus (Adhanom, 2020). Other international authorities also defend this idea through Twitter messages: “Our common enemy is #COVID19, but our enemy is also an ‘infodemic’ of misinformation” (Guterres, 2020).

Rovetta and Bhagavathula (2020) documented several coronavirus fake news widely circulating on the internet, for instance, some people connected to the President of the United States speculating about a “miracle cure” and suggesting an injection of disinfectant could be used to treat the virus. Fernández-Torres et al. (2021) explored through a survey and revision of the literature how the proliferation of COVID-19 false news affects and impacts public opinion in Spain, finding that social networks are considered, by citizens, a source of false news.

Recent research has shown that there has been a significant increase in the belief of conspiracy theories during the COVID-19 pandemic, especially those connected to COVID-19 or anti-vaccination (Leibovitz et al., 2021). During crisis time, with higher levels of stress, anxiety, and uncertainty, conspiracy theories seem to flourish, affecting how individuals behave (Constantinou et al., 2021; Douglas, 2021; Poupart and Bouscail, 2021). In this crisis context, conspiracy theories or narratives play a role in finding meaning, fighting fears, and reducing anxiety (Ali, 2020; Schippers, 2020).

The belief in conspiracy theories has also been considered as a possible factor influencing compliance with the rules of social distance. Conspiracy theories related to COVID-19 have appeared since the beginning of the pandemic, and have been maintained over time (Van Bavel et al., 2020). They reduce the credibility of the official information and could be related to adverse effects regarding compliance with the rules established to avoid contagion. We have been witnesses of a variety of theories regarding the current pandemic: from theories arguing that the Chinese invented the virus, to that it is a biological weapon created by scientists, and to those that Bill Gates intends to use the COVID-19 vaccine to insert a microchip in it and activate 5G technology to take over the world (Ahmed et al., 2020; Sternisko et al., 2020). At the same time, some denialist demonstrations were developed on the international scene (as mass demonstrations in Madrid or Berlin in August 2020, and many other places) suppose risky situations, accompanied by behaviors far from prevention guidelines (Karnitschnig, 2020; Rohde, 2020).

Believing in conspiracy theories associated with COVID-19 poses added risks of worst compliance with social distancing norms. Constantinou et al. (2021) suggested that being highly stressed seemed to increase the probability that a person will believe conspiracy theories, influencing adherence to public health recommendations. Some authors refer to the widespread agreement in the literature that the conspiracy-prone individual is less likely to comply with government recommendations related to handwashing, social distancing, wearing masks, and undergoing diagnostic tests (Poupart and Bouscail, 2021). In addition, a longitudinal analysis showed that people holding more conspiracy beliefs at the beginning of the pandemic showed the lowest increase in social distancing. Other analyses exposed that people who reported more conspiracy beliefs at any wave tended to report less social distancing at the next wave, which poses a significant threat to public health regarding reducing adherence to social distancing measures (Bierwiaczonek et al., 2020). Similar longitudinal findings were recently found regarding the belief in COVID-19 conspiracy theories at the first wave and the resistance to take a vaccine at the second wave (Hornsey et al., 2021).

Other previous articles have already studied some facets of compliance with social distance measures and their effects on the evolution of the pandemic. For example, in Brazil, the study by Oliveira et al. (2020) evaluated the effectiveness of preventive measures regarding the management of the pandemic and highlighted the importance of the involvement and collaborative effort of the entire society in adopting preventive measures against COVID-19 (government, families, and citizens).

Also, research highlights that compliance with social distance measures can be affected by factors such as knowing if there will be a supply, the severity of the sanctions for skipping the measures, support, and trust toward the authorities that issued them. The harshness of the economic and psychological costs of isolation equally affect it (Briscese et al., 2020).

The economic, social, and personal cost paid with isolation is evident, and, logically, citizens want to avoid it. When isolation is done voluntarily or to help others, the costs are lower (Mansdorf, 2020). Briscese et al. (2020), based on a study in Italy, also highlighted that how governments announce social distance measures is relevant for compliance, for instance, regarding the duration of isolation (as it may contrast with expectations of the people), in such a way that if the extensions of confinement are longer than expected, this is associated with a lower predisposition to comply with them. Along the same line, Mansdorf (2020) underlines the importance of providing reliable and accurate information to ensure confidence in the measures related to COVID-19 and the understanding of the threat by the population, to the extent that if there is confidence in the source, greater compliance is expected. In connection with trust, the study by Painter and Qui (2021), based in the United States, reports that the credibility of those who enact social distance measures affects adherence to these policies. It reflects that political beliefs influence the following of the norms related to social distance. Other research suggests the importance of designing health campaigns that emphasize the benefits of protective behaviors rather than debunking misinformation (Hornik et al., 2021).

Previous research on the COVID-19 pandemic has shown essential differences in how countries manage the pandemic, spatial and temporal heterogeneity in COVID-19 spread, and mortality derived from the pandemic (Mansdorf, 2020; Piovani et al., 2021; Yamamoto et al., 2021). Factors associated with better or worse compliance with social distancing norms in the first wave are not identical in all countries. This study aims to understand factors linked to better or worse compliance with social distancing norms in Spain. Thus, the purpose of this study was, on the one hand, to know the Spanish patterns regarding compliance with social distancing measures during the first wave of the pandemic, and on the other hand, awareness of the multiplicity of elements that could affect greater or lesser compliance with social distancing norms. As the bibliographic analysis suggests, we want to identify the importance of different factors associated with compliance with the recommended measures of social distancing in Spain. Likewise, given that there are different social behaviors associated with social distancing, we seek to know if they produce different levels of compliance with the norms of social distance (staying at home, visiting others, shopping, keeping distances, shaking hands, kissing, or hugging). This study would drive a better knowledge of how social distancing norms were complied with in Spain during the most restrictive period of the pandemic, laying the foundations for future preventive actions.

Through the article, for instance, we wanted to know if there is a connection between compliance with social distancing norms and different aspects. Specifically, we wonder if compliance with social distance measures at a very strict lockdown in a country like Spain, where, culturally, social life is wholly linked to sociability in diverse social environments, was associated more with sociodemographic factors, or was the result of a diversity of factors that interplay. Is compliance better understood in the data by sex, age, or other sociodemographic factors than by personal hygiene habits maintained during the pandemic, the support for the political measures established on the pandemic, or the beliefs on conspiracy theories about COVID-19? What is the role of mass media and social media consumption? What is the role of political beliefs, national identification, vote recall, or trust in institutions to understand the observance of norms of social distancing? Do having tested positive for COVID-19 and other aspects connected to physical and psychological health seem important?

This manuscript benefits from statistically representative data concerning how Spanish people declared to behave in the lockdown period. In addition to some sociodemographic factors, it hypothesizes that other variables not so commonly considered in health studies, such as those related to the belief in conspiracy theories, could play a key role in understanding the factors connected to greater or lesser compliance with social distance measures.

## Materials and Methods

### Study Design, Data Collection, and Study Setting

This research, belonging to the *International study on COVID-19* (https://icsmp-covid19.netlify.app/), delves into data collected in Spain from a comparative survey carried out in 67 countries across the world. Some methodological details of this international study can be consulted in Van Bavel et al. (2021, forthcoming).

The Spanish survey is designed to be representative of all people residing in Spain who are over 18 years of age. Valid interviews were obtained from 1,354 participants (1,021 respondents from the Kieskompact Panel 2020, and 333 through contacts on network platforms, such as WhatsApp, email, and Twitter, with the help of the Social Studies and Social Intervention Research Center). The sample invited to take part in the survey was recruited through online Voting Advice Applications developed by Kieskompas (Election Compass) for the Spanish parliamentary elections in 2015 and 2016. Respondents were invited on May 1; a reminder was sent on May 4, 2020. There were 2,000 participants invited: 1,580 started the survey, and 1,107 finished the survey.

The panel is composed based on stratified random sampling, considering four characteristics: gender, age category, educational level, and vote recall. There were 2,000 participants from previous surveys invited to take part in this study. The weighting used in this research makes up 1,090 representative respondents from the Spanish population. The data were weighted based on the Eurostat census, taking into account the age, gender, and educational level of the respondents. The sample was also adjusted to correct for partisan bias through the weighting for vote recall in the 2019 Spanish parliamentary elections. Although under the hypothesis of simple random sampling the error would be +2.9% for global data, as it is a weighted sample, the maximum Wilson margin of error at the 95% confidence level is equal to 7.9. It was calculated with weights incorporated through the effective size of the Kish sample, and with a finite population correction.

**Figure 1:**
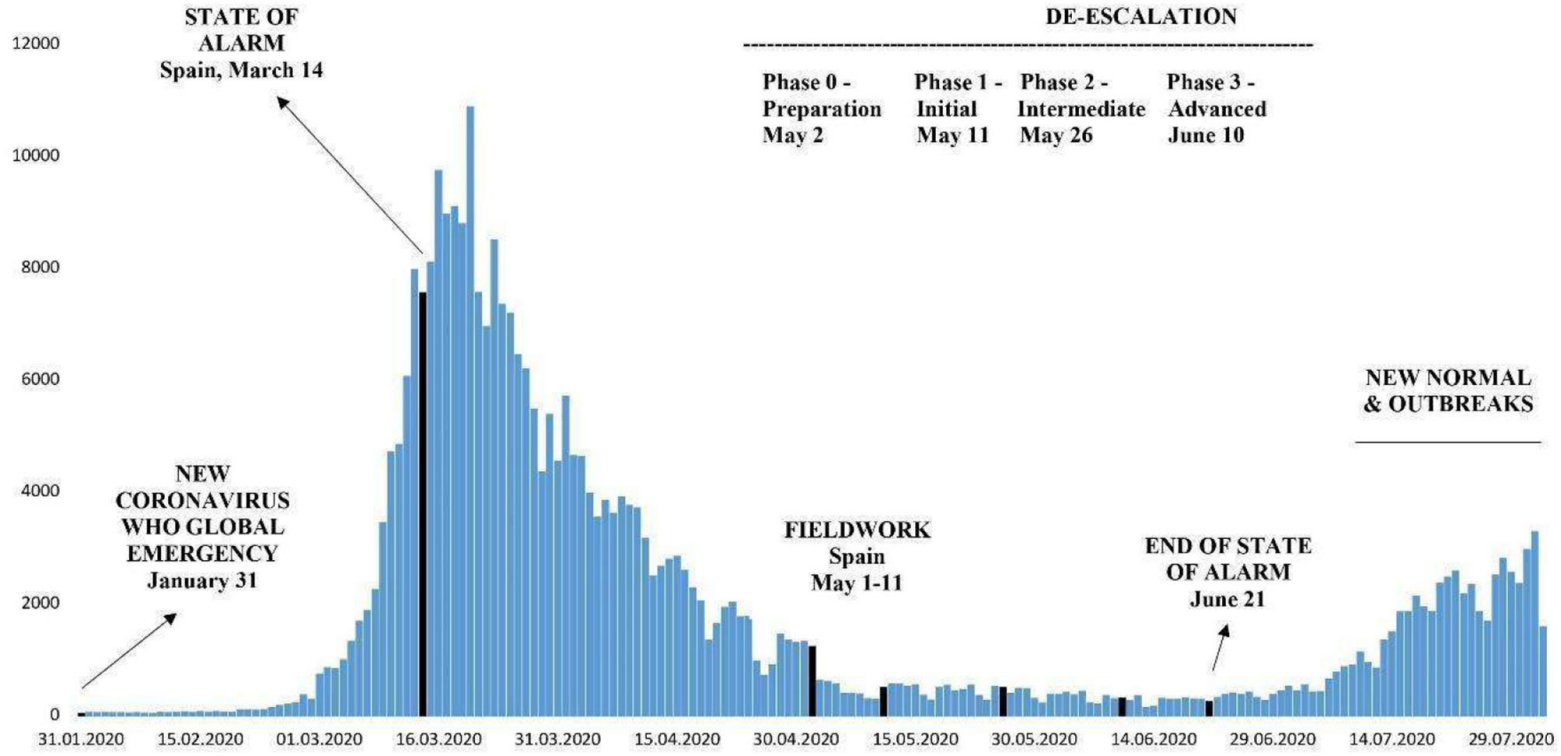
Coronavirus disease-2019 (COVID-19) daily cases in Spain and fieldwork date, 2020. Source: Authors from Ministerio de Sanidad, Consumo y Bienestar Social, cases declared to the Red Nacional de Vigilancia Epidemiológica (RENAVE).

The fieldwork in Spain took place between May 1 and 11, 2020, coinciding with the start of de-escalation for most of the country on May 4 in phase 0 (Figure 1). A pretest of the questionnaire (a pilot study) directed to people of different gender, age, and province was made in Spain by the Spanish team to test if the instrument worked adequately. Also, it was a pretest done by Kieskompas and Vrije Universiteit Amsterdam before the fieldwork as part of their panel survey. The survey was initially sent to a small test sample of 500 respondents on May 1, 2020 as a means of ensuring that there are no display/logic and language issues. Once the data were reviewed, the rest of the sample was invited on that same date.

### Questionnaire, Measures, and Independent Variables

The questionnaire applied in Spain follows the parameters of the international study mentioned above (Van Bavel et al., 2020). For details, the questionnaire can be consulted at the international project website (https://icsmp-covid19.netlify.app/data.html). We used Cronbach's alpha (α) to assess the reliability, or internal consistency, of a set of scale or test items.

Independent sociodemographic variables were gender, age, marital status, number of children, studies, work situation, life satisfaction, and habitat (see descriptives in Supplementary Table 1). Other variables included were habits of personal hygiene maintained during the pandemic (11-point Likert scale from 0 “Strongly disagree” to 10 “Strongly agree;” α = 0.774; mean-based personal hygiene index for regressions); support for established political measures regarding the coronavirus to achieve social distancing (11-point Likert scale from 0 “Completely disagree” to 10 “Completely agree”; α = 0.904; index of support for policies regarding coronavirus based on the mean for the regressions). A variable was created to collect the terms of the interaction between the previous ones, hygiene and support for policies.

Likewise, we included the five-item Conspiracy Mentality Questionnaire (CMQ; Bruder et al., 2013), measured on a 11-point scale from 0 (“Not at all, 0%”) to 10 (“With total certainty”, 100%; α = 0.843); for example: “I think... -... many important things happen in the world that people are not informed about”). Also included were four items relating to conspiracy theories associated with COVID-19, measured on an 11-point scale from 0 (“Completely disagree”) to 10 (“Completely agree”; α = 0.893); for example: “The coronavirus (COVID-19) is a bioweapon engineered by scientists”).

On the other hand, to evaluate whether there were differences concerning the frequency of use to obtain information through different means (television, paper, newspapers, magazines, radio, online digital newspapers, blogs, and social networks), seven variables were employed (specific of the Spanish study) that were significant in previous related studies (Gualda et al., 2019; Rodríguez-Pascual et al., 2021): “How often do you use the following means to inform yourself”: 1 (“Never”) to 6 (“Every day”), constructing two indices with variables previously grouped through cluster analysis: summation index of frequency of use to obtain information through modern means and summation index of frequency of use to obtain information through four traditional media.

We used a political self-identification scale (0-Very left, 10-Very right) and political and institutional trust variables: vote recall in the general elections of 2019, the degree of trust toward sixteen institutions and organizations, specific of the Spanish study, (Do you trust the following institutions/organizations?; with a 4-point scale (1 “No, not at all”, 4 “Yes, totally”), constructing an index of trust in institutions and organizations based on the mean (α = 0. 834), and an index of national identification based on the mean of two-item (0–10).

The questionnaire incorporated four variables related to the COVID-19 situation. Two related to risk perception: “By April 30, 2021: How likely do you think it is that [1-you] [2-the average person in Spain] will get infected by the Coronavirus (Covid-19)?” (0–100 scale). Furthermore, two others that measured the experience of having tested positive for COVID-19: “Have you tested positive for the Coronavirus (COVID-19), meaning that you (now or earlier) have had a medically confirmed case of this disease?;” “Has anyone you know well (friend, partner, family, colleague, etc.) tested positive for the Coronavirus (COVID-19)?”

We employed variables related to physical and psychological health: health: “In general, how would you rate your physical health as it is today? (0-Extremely bad, 10-Extremely good);” happiness: “In general, to what extent do you feel happy these days?” (0-Very unhappy; 10-Very happy); self-esteem: “I have high self-esteem” (0-Strongly disagree, 10-Strongly agree; from Robins et al., 2001); life satisfaction, from the Cantril scale (“Please imagine a ladder, with steps numbered 0 at the bottom and 10 at the top. The top represents the best possible life for you, and the bottom represents the worst possible life for you. On which step of the ladder would you say you personally feel you stand at this time?,” adapted from Bjørnskov (2010); future optimism (optimistic trait): “As a person, I am always optimistic for my future” and “Overall, I expect more good things will happen to me than bad” (both on a scale from 0-“Strongly disagree” to 10-“Strongly agree”).

### Dependent Variables

Six dependent variables measured the behavior of Spaniards during the pandemic regarding their compliance with the measures of social distance, particularly behaviors such as staying at home, visiting other people, frequency of purchase, maintenance of distances, and habits such as shaking hands or kissing and hugging outside the home (0-Completely disagree, 10-Completely agree). The models explained below use these dependent variables.

### Hierarchical Multiple Regression Models: Procedure and Analytical Strategy

**Figure 2:**
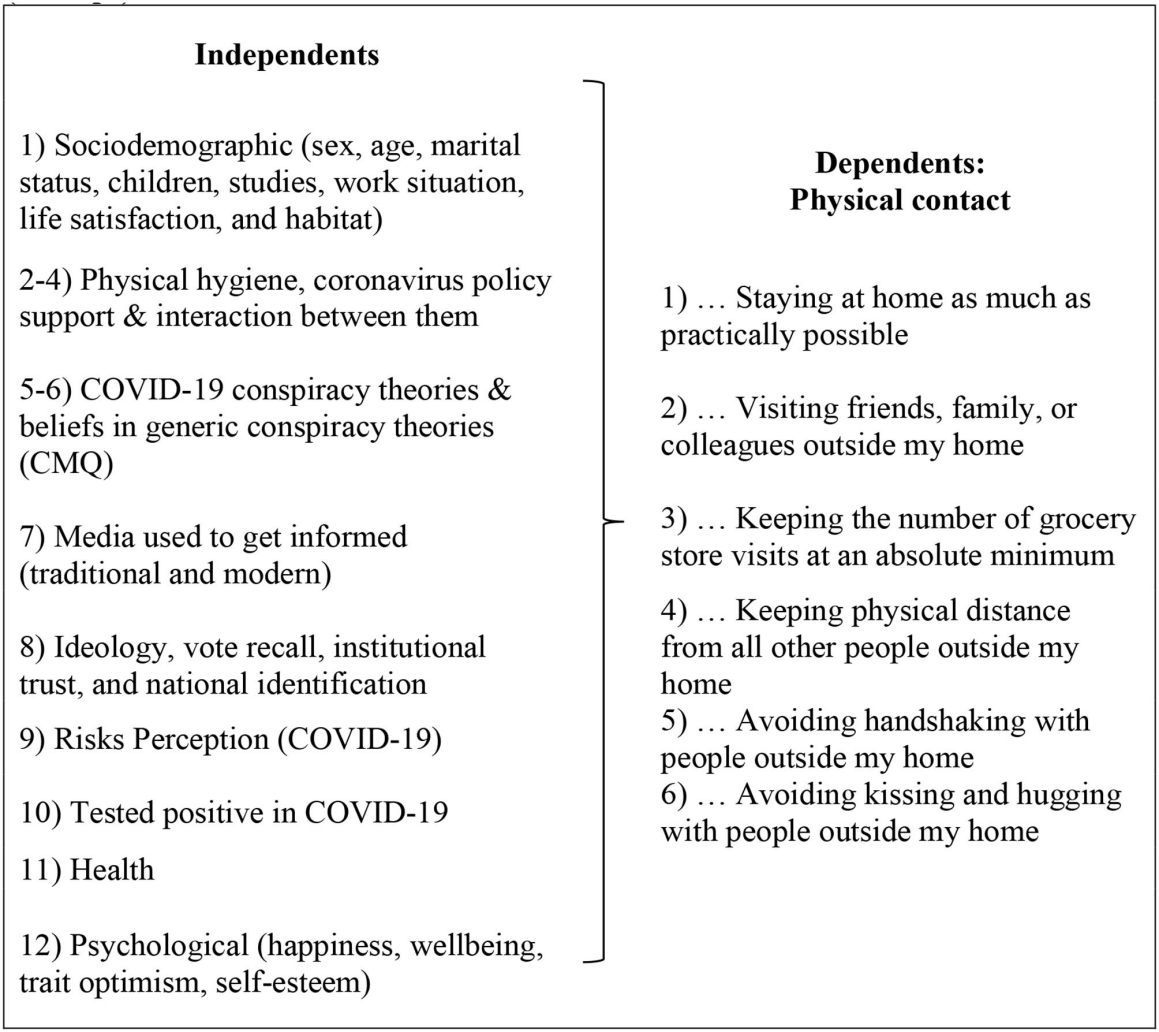
Dimensions and variables included in the six hierarchical multiple regression models (12 steps). Source: Authors.

The objective of the analyses was to analyze the relationship between the six dependent variables mentioned above and social distancing. For this, the six hierarchical multiple regression models are calculated following the scheme shown in Figure 2. A univariate and bivariate exploratory analysis was carried previously. It considered the relationship between each dependent and the independent variables, such as bivariate linear regressions with each dependent variable related to social distance. The quantitative variables in the analysis were standardized as z scores (zero mean and standard deviation 1). Given that the six dependent variables were continuous or metric, hierarchical multiple regression was chosen to evaluate the effect that different studied dimensions contributed to the models. Variables that contributed to multicollinearity problems or that were not significant in different preliminary analyses were excluded.

The assumptions and requirements for this type of multiple linear regression model were checked (size of the sample, metric-dependent variable, relevant independent variables, linearity, normality) (Cea D'Ancona, 2002; Field, 2018). Different actions were performed in the process of checking assumptions that we have summarized here:

- Elimination of non-relevant variables for the model when they did not contribute to the models increasing *R*^2^ significantly (bivariate correlations, partial regressions, and changes in R^2^ were checked for the six models).
- Categorical or qualitative independents variables were previously transformed as dummy variables to comply with the requirement of being metric.
- Standardization in z-scores to neutralize the units of measurement.
- Linearity of the models was checked visually through partial regression plots and standardized residual plots.
- Outliers were checked.
- Normality was examined through different procedures (histograms, boxplots, Q–Q plots, and tests). It was checked qualitatively (graphical approach) that histograms of standardized residuals were normally distributed. Also, Q–Q plots were examined. It was also checked the skewness and kurtosis for each variable included in the model. Due to the scarce utility of the Kolmogorov–Smirnov test (Lilliefors) in big samples (>1,000) to test the null hypothesis that a set of data comes from a normal distribution, and the limitations of the Shapiro–Wilk test for big samples (Cea D'Ancona, 2002; Field, 2018), although they were calculated, we trusted more on graphic approximation and skewness, and kurtosis. Although the Shapiro–Wilk test is reported to be better than Kolmogorov–Smirnov, and it also works for small and large samples (Razali and Wah, 2011), some authors recommend graphical approaches and the study of skewness and kurtosis to evaluate normality for the samples size (Cea D'Ancona, 2002; Field, 2018).

In the case of non-normality through visual or skewness and kurtosis approaches, in Supplementary Material, Supplementary Table 1 with descriptives, we included median and interquartile ranges (IQRs).

## Results

### Univariate Analysis

The univariate analysis shows that most Spaniards declared that they adequately followed the social distance measures established during the pandemic [State of Alarm phase]. On a scale of 0–10 (Table 1), on average, 9.78 Spaniards indicated that they had avoided kissing or hugging outside the home. Very few visited friends, family, and co-workers outside the home (9.4), and almost all avoided shaking hands outside the home (9.73) or stayed home as much as possible (9.5). Also, a significant part of the Spaniards kept their distance with everyone outside their homes (9.39), and a large part of those interviewed tried to go shopping as little as possible (8.75).

**Table 1:** Physical contact: be as precise as you can, during the pandemic (COVID-19).

	**Mean**	**SD**
(1) … Staying at home as much as practically possible (*n* = 1,090)	9.5	1.06
(2) … Visiting friends, family, or colleagues outside my home (*n* = 1,090)	9.5	1.06
(3) … Keeping the number of grocery store visits at an absolute minimum (*n* = 1,083)	8.75	2.29
(4) … Keeping physical distance from all other people outside my home (*n* = 1,089)	9.39	1.37
(5) … Avoiding handshaking with people outside my home (*n* = 1,089)	9.73	1.29
(6) … Avoiding kissing and hugging with people outside my home (*n* = 1,089)	9.78	1.19

*Source: Authors. 0-Completely disagree, 10-Completely agree (Item 2 was inverted from the original questionnaire)*.

According to the standard deviations, there is a certain lower degree of compliance with the rules and a greater disparity with the rest of the items concerning purchases away from home (SD 2.29). Nevertheless, globally, the high level of compliance with the pandemic guidelines during the State of Alarm is striking, considering the importance of physical proximity, shaking hands, hugging, or kissing in Spanish culture.

### Multiple Linear Hierarchical Regressions

A series of bivariate linear regressions were carried out to delve into the factors explaining greater compliance with social distance rules, and to verify the most suitable variables to incorporate in the hierarchical multiple regression models. The hierarchical multiple regression models were created in order to elucidate whether, for the six dependent variables considered relative to personal contact (1-staying at home, 2-visiting away from home, 3-shopping, 4-keeping distance, 5-shaking hands, 6-kissing or hugging), each dimension studied either contributed or not to a significant change in the explanation of the variance. Not all the six behaviors (dependents) that were measured had to play the same role in this regard.

To use the six regression models, the variables were grouped into 12 different dimensions to be entered step by step in SPSS (Method = Enter) (see analytic schema in Figure 2). These models were able to explain a significant percentage of the variance based on the contributions of different dimensions and variables. Thus, we found that compliance with the social distance norms has a multifactorial explanation, with statistically significant variances at explaining the variance in the six dependent variables (F accomplishes the requirements to be significant in all the models). According to the most explanatory value (percentage of explained variance, *R*^2^), the respondents in Spain have tried to go shopping as little as possible (*R*^2^: 49.1%), have stayed home as much as possible (*R*^2^: 43.9%), have kept a distance with everyone outside their home (*R*^2^: 32.6%), have avoided kissing or hugging outside their home (*R*^2^: 29.3%), have avoided shaking hands outside their home (*R*^2^: 23.1%), and have visited friends, family, and work colleagues outside their home (*R*^2^: 20.4%) (Table 2).

**Table 2:** Hierarchical regression models and statistics of change.

	**(1) … Staying at home as much as practically possible**			**(2) … Visiting friends, family, or colleagues outside my home**			**(3) … Keeping the number of grocery store visits at an absolute minimum**			**(4) … Keeping physical distance from all other people outside my home**			**(5) … Avoiding handshaking with people outside my home**			**(6) … Avoiding kissing and hugging with people outside my home**		
**Steps**	***R*** ^**2**^	**Change in R squared**	**Sig. Change in F**	***R*** ^**2**^	**Change in R squared**	**Sig. Change in F**	***R*** ^**2**^	**Change in R squared**	**Sig. Change in F**	***R*** ^**2**^	**Change in R squared**	**Sig. Change in F**	***R*** ^**2**^	**Change in R squared**	**Sig. Change in F**	***R*** ^**2**^	**Change in R squared**	**Sig. Change in F**
1. Sociodemographic	0.178	0.178	0.000	0.107	0.107	0.000	0.206	0.206	0.000	0.096	0.096	0.000	0.058	0.058	0.000	0.057	0.057	0.000
2. Physical hygiene	0.220	0.042	0.000	0.112	0.005	0.022	0.252	0.045	0.000	0.169	0.073	0.000	0.125	0.066	0.000	0.129	0.072	0.000
3. Coronavirus policy measures support	0.233	0.013	0.000	0.112	0.000	0.837	0.253	0.002	0.153	0.182	0.013	0.000	0.129	0.004	0.030	0.135	0.007	0.005
4. Interaction between physical hygiene and policy measures support	0.322	0.090	0.000	0.119	0.007	0.004	0.259	0.006	0.005	0.204	0.022	0.000	0.146	0.017	0.000	0.189	0.054	0.000
5. COVID-19 conspiracy theories	0.324	0.002	0.645	0.131	0.012	0.007	0.363	0.104	0.000	0.223	0.019	0.000	0.159	0.013	0.004	0.217	0.028	0.000
6. Beliefs in generic conspiracy theories (CMQ)	0.377	0.053	0.000	0.142	0.011	0.030	0.384	0.021	0.000	0.242	0.019	0.000	0.171	0.012	0.014	0.230	0.014	0.003
7. Mass Media and Social Networks used to get informed (traditional and modern)	0.394	0.017	0.000	0.151	0.009	0.004	0.384	0.000	0.822	0.254	0.012	0.000	0.178	0.008	0.010	0.239	0.008	0.004
8. Ideology, vote recall, institutional trust, and national identification	0.421	0.026	0.000	0.175	0.024	0.001	0.470	0.086	0.000	0.304	0.049	0.000	0.209	0.031	0.000	0.268	0.029	0.000
9. Risks Perception (COVID-19)	0.421	0.001	0.653	0.179	0.004	0.092	0.473	0.004	0.035	0.306	0.002	0.202	0.215	0.006	0.018	0.278	0.010	0.001
10. Tested positive in COVID-19	0.424	0.002	0.135	0.179	0.000	0.799	0.474	0.000	0.749	0.315	0.010	0.001	0.215	0.000	0.973	0.284	0.006	0.015
11. Physical Health	0.424	0.001	0.228	0.179	0.000	0.998	0.474	0.000	0.707	0.315	0.000	0.873	0.215	0.000	0.608	0.284	0.000	0.812
12. Psychological	0.439	0.015	0.000	0.204	0.024	0.000	0.491	0.018	0.000	0.326	0.011	0.007	0.231	0.016	0.001	0.293	0.009	0.038

*Source: Authors*.

Table 2 reports the statistics of change and the significance of the change in F in hierarchical regression. Steps 1, 2, 4, 6, 8, and 12 showed that sociodemographic variables, personal hygiene behaviors, and interaction between personal hygiene behaviors and support for political measures have significantly contributed to explaining the variance of the six dependent variables related to the observance of social distancing. Also, this is the case with conspiracy mentality, political ideology, vote recall, trust in institutions, national identification, and psychological factors.

Besides, five of the six dependent variables related to personal contact patterns in the pandemic were also explained by dimensions regarding the belief in specific conspiracy theories related to COVID-19, variables associated with mass media consumption, or social networks used to get information. Support for political measures enacted during the pandemic also acted as a significant explaining factor in four of the six dependent variables.

The variable physical health did not contribute to explaining important changes in the variance of the six dependent variables corresponding to the patterns followed by social distance during the pandemic. The rest of the 12 dimensions considered not yet cited contributed to the explanation of at least two or three of the six dependent variables (perception of risks and testing positive for COVID-19).

Regarding specific variables that acted as explicative of compliance with the six measures of social distance, we found that the explaining factors were not the same for the six dependent variables of social distance. Globally, focusing on significant standardized beta coefficients (see Supplementary Material for details, Supplementary Table 2), the positive coefficients indicate an association with greater compliance with the measures of social distance. In contrast, negative coefficients refer to worse compliance. In general terms (sometimes only regarding some of the six dependent variables with significant beta coefficients), better compliance with social distancing norms was found in the case of females, older, respondents with a low level of education than postgraduates; and employed, unemployed, and students. Significant beta coefficients were not found for each of the six dependents considered. Therefore, in this section, we only summarize what is referred to significant coefficients, independently if they are found for one to six of the dependent variables. For instance, being older is associated with more compliance only with regard to keeping the number of grocery store visits at an absolute minimum.

The observance of personal hygiene measures, the support for policies regarding the coronavirus, trust in institutions and organizations, national identification, the consumption of modern means of information (online digital newspapers, blogs, and social networks), life satisfaction (Candril scale), and self-esteem were positively associated with the following of social distancing norms.

Negative coefficients, representing worse compliance, were found in single people compared with married ones, the highest number of children, the interaction between the observance of personal hygiene measures and support for policies regarding the coronavirus. Also, the consumption of traditional means of information (television, paper, newspapers, magazines, and radio), and a higher level of happiness helped to explain worse compliance.

Different items related to conspiracy mentality (Conspiracy Mentality Questionnaire) were associated with both better and worse compliance with social distancing norms. Positive coefficients were found concerning beliefs in this scale as “… many very important things happen in the world, which the public is never informed about”, and “…events which superficially seem to lack a connection are often the result of secret activities”. In contrast, negative coefficients or worse compliance was connected to other beliefs as “… politicians usually do not tell us the true motives for their decisions” and “… there are secret organizations that greatly influence political decisions”.

On the other hand, the belief in specific COVID-19 conspiracy theories also helps to explain the compliance with social distancing norms. Better compliance is associated with beliefs such as: “The coronavirus (COVID-19) is a hoax invented by interest groups for financial gains” and “The coronavirus (COVID-19) was created as a cover up for the impending global economic crash.” On the contrary, worse observance is found in those that believe in the idea “The coronavirus (COVID-19) is a conspiracy to take away citizen's rights for good and establish an authoritarian government.” Nevertheless, positive and negative beta regression coefficients were found depending on the dependent variable concerning “The coronavirus (COVID-19) is a bioweapon engineered by scientists.”

The tendency to better compliance with social distancing norms is higher among those with a left-wing ideological outlook, and considering vote recall, in political parties associated with the left such as the Socialist Party (PSOE), and Unidas Podemos (left), who were governing Spain at the time of this study. On the contrary, vote recall to more conservative political parties in Spain (PP and Ciudadanos) explains less compliance with social norms.

About other specific dimensions (or variables) considered in the analysis, we found positive or negative beta regression coefficients depending on the dependent variable that was considered. That was the case of variables such as habitat (urban), optimism, or how the respondents place themselves on a ladder to represent if they think they stand at the top or the bottom compared with other people in Spain.

## Discussion and Conclusions

### Sociodemographic Factors

This study shows the multifactorial nature that necessarily includes the explanation of the guidelines for compliance with social distance measures in Spain during the COVID-19 pandemic. Although most of the Spaniards declared that they had followed social distancing measures during the state of alarm (Table 1), in a similar way that happened in other countries in wave 1, some of the variables were more associated than the others with less or higher compliance with these norms. Among the most noteworthy aspects, we discovered that various dimensions contribute to explain in the data the fulfillment of social distance measures in the six dependent variables considered. Some patterns show the importance of sociodemographic variables (such as higher compliance among women, older people, and citizens with lower levels of education; or the inverse trend in single people and a higher number of children). The higher compliance with personal hygiene measures and the support for the political measures ordered by the government related to COVID-19 was also associated with higher compliance with social distancing norms. These results are connected to other international studies where sociodemographic factors help to explain compliance with social distancing measures or intentions to comply in different countries (Xu et al., 2020; Smith and Branscum, 2021; Uddin et al., 2021). Coherent with this research, other studies report that younger people are found less inclined to follow social distancing norms (Margraf et al., 2020; Xu et al., 2020; Smith and Branscum, 2021).

Mansdorf (2020), for example, elaborated on a typology that delimits refusers, deniers, and young people. Refusers are those who openly oppose following the rules, knowing that they create risks for others or those who think they are not going to be infected. Deniers, those who have a personality and social identity closely linked to social interaction, see their routines altered and demand return to normality. Young people are those who seem to have a less developed sense of responsibility. Concerning this idea of social or civic responsibility, after a first wave in which COVID-19 cases and deaths were exceptionally high between older people in Spain, we have assisted to a rejuvenation of the profile of infected cases, highly associated with relaxation of behaviors of social distancing between younger people.

### Ideology, Voting, and Trust

Regarding ideology and voting, we found substantial effects and significant beta regression coefficients explaining compliance in this study. First, regarding political ideology, there seems to be a tendency among the ideologically left-wing Spanish to be more inclined to comply with social distance norms. The opposite occurs with the right-wing respondents. This tendency is statistically significant regarding the compliance of norms as staying at home and keeping the number of grocery store visits at an absolute minimum (Supplementary Material, Supplementary Table 2).

Nevertheless, the patterns seem not always so clear when we explore vote recall in Spain. Regarding vote recall (PSOE, Socialist party as reference variable for regression models), as there seems to be a tendency among those who voted for center-right political parties (PP and Ciudadanos) to be less inclined to comply with social distancing norms. The opposite occurs with those located more to the left (PSOE and Unidas Podemos). Nevertheless, going against the expected behavior according to ideology, VOX voters declared better compliance with social norms than the other respondents who declared having voted for the right or center (PP and Ciudadanos).

Considering that the government of Spain was composed of a coalition of left-wing parties during the pandemic, this pattern, except for VOX, is coherent with ideological self-identification. However, among the attributable explanations necessary to delve into further studies, there may be diverse factors such as the sociodemographic profile of voters (gender and age), the political specificities of Spain, conservatism patterns directed to follow preventive social distance guidelines, and others.

Other international studies have also found partisan differences related to social distance in response to the coronavirus, but with an axis on mobility and geolocation indicators, GPS, and sources other than the survey (Allcott et al., 2020; Andersen, 2020; Barrios and Hochberg, 2020; Engle et al., 2020). The complexity involved in managing the COVID-19 pandemic becomes evident when considering the factors that are associated with better or worse compliance with social distance measures, having already detected diverse sociological profiles in other countries that pose a threat to the whole, by not following the measures provided by the government.

Regarding trust in institutions, we have found significant beta coefficients in four of the six regression models explaining the compliance of social distancing measures. The results have pointed at the idea that higher trust is associated with better compliance with social distancing norms. Other studies have revealed that trust in government or institutions is a significant factor in some countries (Travaglino and Moon, 2021; Uddin et al., 2021). On the other side, some studies suggest that public trust in governments and institutions mediates adherence to policy restrictions during the COVID-19 pandemic (Pak et al., 2021). In a country like Spain, where credibility and trust in some political institutions are not very high, it would be a fundamental challenge for future actions.

### Conspiracy Theories, Misinformation, and Communication

The results suggest that the belief in conspiracy theories seems to be one of the factors more strongly associated with compliance with social distancing norms in Spain during the first stage of the COVID-19 pandemic. Nevertheless, as for the results, it seems to operate non-univocally according to the dependent variable considered. The belief in conspiracy theories is one of the determinants that may have captured more attention during this COVID-19 pandemic. A proposal for future investigations, derived from the results of this study, is to go deep into elements related to the current infodemic (conspiracy theories or another kind of misinformation). As argued by Poupart and Bouscail (2021), this pandemic demonstrates that the spread of false news and support to conspiracy theories is not marginal, which could bring potentially negative impacts in terms of public health. Other studies have highlighted the risks to public health that a segment of the population supports the fake news and misinformation circulating through social media (Cuan-Baltazar et al., 2020; Van Bavel et al., 2020). Other authors have also paid attention to social media exposure and the influence on preventive behavior in COVID-19 (Nazir et al., 2020). Although, as they highlight, the bibliography is still scarce regarding the social aspects of COVID-19, having identified the factors that explain greater compliance with social distance measures has been critical in this article. Nevertheless, it is essential to be conscious of the variety of facilitators of adherence to social distancing measures and the difficulties of finding perfect compliance (Coroiu et al., 2020).

### Limitations, Conclusions, and Future Research and Actions

Finally, this cross-sectional research has sought to explore the associations between different factors and compliance with the rules of social distance during the first wave of the COVID-19 pandemic in Spain. To the extent that this study is not longitudinal, interviewing the same people at different times of the pandemic, it is not feasible to establish causal relationships based on behaviors that are based on temporal logic. This study does not provide information on cause-and-effect relationships, because it offers a portrait of a single moment in time (first wave of the COVID-19 pandemic). Also, the sample is not broad enough to disaggregate results between the different regions in Spain. However, it allows offering results relative to the patterns of Spain as a whole, as a specific place and sociocultural context in which the pandemic has developed. Subsequent and current studies in Spain could be able to overcome these limitations. Also, other longitudinal studies will be able to test other causal hypotheses, which this study could not explore.

In this study, the multiple regression models principally predict the values of a dependent or criterion variable from the other independent variables or predictors, looking for a model that better explains the dependent (Field, 2018). In this case, six criterion variables representing different social distancing measures were considered (six models). On the other hand, the explanatory capacity reached by some variables in the models suggested some lines of exploration for further research. A better understanding of factors that help improve compliance with health norms and the knowledge of the elements that better explain the observance of health norms are essential for the prevention, reduction of infections, and reduction of mortality during pandemics.

On the other hand, one of the main strengths of this research is that it has investigated the multiplicity of factors that help to understand compliance with social distance guidelines in a Latin country characterized culturally by sociability based on the high proximity of people. Knowing what affects compliance with the recommended distance guidelines in an epidemic situation would provide us with data of interest compared with other equivalent contexts, taking into account that voluntary personal choices, emotions, and various types of risk perceptions are factors that have to do with behavior and whose intervention can be addressed.

The information obtained provides valuable data for public health management. This information is helpful to prevent new outbreaks and to help slow down the epidemic curve. Identifying sociological profiles more or less prone to comply with the rules of social distancing, and knowing the variables that are significant in the multifactorial explanation of this observance of rules are an essential help for managers in this field. Concerning the COVID-19 pandemic, this study supports the idea that the foundations of public health governance are being shaken internationally (Cori et al., 2020), connected to the necessity to expand the measurement of the classic determinants of health (Khubchandani et al., 2020). This extension implies considering other variables in health research that connect with the development of the perceptions of the population of risk, social media, the infodemic, or even the dissemination of conspiracy theories.

Apart from considering old and new explaining factors, it is also important not to forget the influence of public institutions and government in managing the pandemic. Some relevant Spanish scientists have suggested that after Spain became “one of the worst affected countries,” an independent and impartial evaluation of this crisis is required, an evaluation focused “on the activities of the Central Government and of the governments of the 17 autonomous communities,” such as the evaluation of “governance and decision making, scientific and technical advice, and operational capacity” (García-Basteiro et al., 2020, p. 596).

Other lessons for the future that this pandemic has brought in Spain are the importance of focusing on short-term actions and designing strategies to follow in the medium-and longer-term. Here, reflecting on the patterns of Spanish sociability, similar to those in other Latin American or Mediterranean countries, we consider it extremely important to design preventive policies destined to counteract the effects of long confinements on social and family relationships. It is also of great relevance anticipating measures destined to prevent adverse effects on the economy, mental health, or even directed to specific vulnerable collectives as children.

Public health also needs to study how to involve families, friends, or even other people or organizations to promote better compliance in the long-term when lockdown measures are relaxed. The community must perceive it as a healthy asset and positively value the protective measures so that they can be integrated as a habit. In addition, there is a need to develop actions directed to individuals less prone to trust in government and institutions to prevent better compliance measures, primarily when after states of alarm, subsequent phases of a pandemic rely on social or civic responsibility.

The results of this study concerning the endorsement of conspiracy theories support the idea of considering, for future policies, the different faces of public communication, such as those connected to an improvement of the perception of governments. Positive perceptions seem to be among the factors associated with the highest adherence to social distancing norms (Margraf et al., 2020). Also, as for the suggestion of Hornik et al. (2021), it seems necessary to emphasize, in public communications, the benefits of protective behaviors.

Finally, the data suggest that compliance with social distance during the first wave in Spain has a multifactorial explanation, suggesting that proper management of the pandemic should incorporate medical interventions and address other aspects such as those linked to social communication processes. Thus, in current times, the importance of developing information reinforcement measures cannot be underestimated. This research found factors explaining less compliance to norms, some of which are connected to new social realities, even when we discovered that most Spaniards were compliant with the established guidelines of social distance during the first wave.

Highlighting this dimension, a social context where disinformation and conspiracy theories around COVID-19 has increasingly developed, as in Spain, suggests to researchers the importance of exploring the role of new communication patterns, new media, or social behaviors connected to them. Minimizing the adverse effects on public health of novel dimensions analyzed in this study is a path to consider in further research. The detection of social profiles less compliant with social distance measures (although most of the population followed the recommendations) is of help in the face of new outbreaks or coming epidemics, as in the case of Spain several months after this fieldwork, evidenced.

Furthermore, the results allow us to suggest that health management should spare no effort in combating the development of fake news, disinformation, and conspiracy theories around the pandemic. Its influence on the compliance or not with the standards necessary to reduce health collapse and the socioeconomic implications of the pandemic make it essential not to underestimate its importance for public health in the current context. Revitalized anti-vaccine, negationist, or conspiratorial movements are dangerous enough to promote their neutralization.

Nevertheless, in addition to these factors, this research concludes that there is a diversity of elements that are related to the compliance with social distance norms, such as sociodemographic ones, which suggests that global reductionist explanations may fail. In conclusion, we consider knowing the patterns and country particularities of compliance with recommended distance guidelines in an epidemic situation. Besides, the location provides data of interest compared with other similar sociocultural contexts in the world. Further research may explore and continue to test these findings in other locations and periods of time.

## Data Availability Statement

The original contributions presented in the study are included in the article/Supplementary Material, further inquiries can be directed to the corresponding author.

## Ethics Statement

The study was approved in Spain by the Andalusian Government, Ethic Committee for Biomedical Research in Andalusia (1175-N-20). The international Project in which it is framed was also approved by the University of Kent, number of approval: 202015872211976468. The participants gave written informed consent to participate, and the data was kept confidential. The participants provided their written informed consent to participate in this study.

## Author Contributions

EG was responsible for coordination, designing the article, analysis, interpretation of data, and writing the manuscript. MPG participated in the analysis. MPG, EMM, IRP, and EBGN participated in the interpretation of data and writing the manuscript. All the Spanish researchers participated in the translation of the questionnaire into Spanish, and in the development of a pre-test. AK was responsible for data collection at Kieskompas, with the help of the rest of the researchers. All the authors read and approved the final manuscript.

## Funding

Funding for open access charge: University of Huelva, *Social Studies and Social Research Center* [SEJ-216], and COIDESO (Spain). Kieskompas (Election Compass) and Vrije Universiteit Amsterdam (The Netherlands) supported the fieldwork.

## Conflict of Interest

The authors declare that the research was conducted in the absence of any commercial or financial relationships that could be construed as a potential conflict of interest.

## Publisher's Note

All claims expressed in this article are solely those of the authors and do not necessarily represent those of their affiliated organizations, or those of the publisher, the editors and the reviewers. Any product that may be evaluated in this article, or claim that may be made by its manufacturer, is not guaranteed or endorsed by the publisher.
