# Supplementary material for: Social Distancing and COVID-19: Factors Associated With Compliance With Social Distancing Norms in Spain
Source: Front Psychol. 2021 Sep 14;12:727225. doi: 10.3389/fpsyg.2021.727225 (PMC8476756; doi:10.3389/fpsyg.2021.727225)
Supplement: Supplementary file 1 [file Table_1.pdf]

Supplementary Material

Table S1. Sociodemographic characteristics of the sample

	**Total (N=1090)**<br>n (%)/ Mean (SD)	**Missing values**<br>n (%)
**1) Sociodemographic**		
Female	51.7 (47.4)	0
Age	46.25 (SD 15.74)	0
Single, without a partner	284 (26.4)	15 (1,4)
Single in a relationship	296 (27.1)	
Married	495 (45.4)	
Number of children	0.93 (1.05)	
Primary	38 (3.5)	0
High school	381 (34.9)	
Vocational training	329 (30.2)	
University (Degree, Bachelor)	173 (15.9)	
University (Master, Doctorate)	168 (15.4)	
Employed full time	548 (50.3)	0
Employed part-time	73 (6.7)	
Unemployed / Looking for work	108 (9.9)	
Student	94 (8.6)	
Retired	196 (18)	
Other	71 (6.5)	
Where would you place yourself on this ladder to represent where you think you stand at this time in your life, compared to other people in Spain? (10-At the top, 0-At the bottom	5.15 (SD 1.66)	1 (0,1%)
Habitat: Urban	894 (82)	0
Physical hygiene and support for political measures regarding the coronavirus		
**2)** Average based personal hygiene index (0-10)	7.78 (SD 1.99)	0
**3)** Coronavirus policy support index based on Mean (0-10)	8.59 (SD 2.15)	0
Median (IQR)	0.33 (0.70)	
**4)** Interaction Personal hygiene * Policy support regarding coronavirus (0-100)	67.94 (SD 25.79)	0
Median (IQR)	0.11 (0.63)	

Table S1. Sociodemographic characteristics of the sample (cont.)

	Total (N=1090)<br>n (%)/ Mean (SD)	Missing values<br>n (%)
**Conspiracy theories**		
5) Specific COVID-19 conspiracy theories		
The coronavirus (COVID-19) is a bioweapon engineered by scientists (0-10)	3.62 (SD 3.39)	0
The coronavirus (COVID-19) is a conspiracy to take away citizen's rights for good and establish an authoritarian government (0-10)	2.85 (SD 3.16)	0
The coronavirus (COVID-19) is a hoax invented by interest groups for financial gains (0-10)	2.04 (SD 2.85)	0
The coronavirus (COVID-19) was created as a cover up for the impending global economic crash (0-10)	2.36 (SD 2.92)	0
6) Beliefs in general conspiracy theories (CMQ). I think… 1-Certainly not 0%, 11-Certain 100%)		
… many very important things happen in the world, which the public is never informed about	8.51 (SD 2.45)	7 (0,6)
… politicians usually do not tell us the true motives for their decisions.	8.59 (SD 2.29)	26 (2,4)
… government agencies closely monitor all citizens	6.30 (SD 2.82)	17 (1,6)
… events which superficially seem to lack a connection are often the r0esult of secret activities.	6.01 (SD 2.89)	19 (1,8)
… there are secret organizations that greatly influence political decisions.	6.43 (SD 3.05)	24 (2,2)
**Mass media and social networks used to get informed**		
**7)** Summation Index of the frequency of use of 3 modern means to get informed (online digital newspapers, blogs, social networks as Facebook, Twitter, Instagram, etc.) (3-18)	11.81 (SD 3.08)	11 (1.1)
Summation Index of the frequency of use of 4 traditional means to get informed (television, paper newspapers, magazines, radio) (4-24)	12.22 (SD 3.97)	30 (2.8)

Table S1. Sociodemographic characteristics of the sample (cont.)

	**Total (N=1090)**<br>n (%)/ Mean (SD)	**Missing values**<br>n (%)
**Ideology, vote, and trust in institutions**		
**8)** Overall, how would you describe yourself in terms of political ideology? (0-10)	4.00 (SD 2.61)	0
**Vote recall**		
PSOE	219 (20.1)	0
PP	122 (11.2)	
VOX	114 (10.4)	
Unidas Podemos	96 (8.8)	
Ciudadanos	54 (5.0)	
Others	152 (14.0)	
Did not vote	334 (30.6)	
Index of Trust in institutions and organizations based on the average of 16 institutions (1-4)	2.48 (SD 0.46)	7 (0.6)
Index of National Identification based in the mean of two-item (0-10)	5.34 (SD 3.01)	1
**Risk perception and testing positive for COVID-19**		
**9)** By April 30, 2021: How likely do you think it is that you will get infected by the Coronavirus (Covid-19)? (0-100)	49.91 (SD 26.82)	0
By April 30, 2021: How likely do you think it is that the average person in Spain will get infected by the Coronavirus (Covid-19)? (0-100)	56.73 (SD 23.85)	5 (0.4)
**10)** Have you tested positive for the Coronavirus (COVID-19), meaning that you (now or earlier) have had a medically confirmed case of this disease?		
NO	1077 (98.8)	1 (0.1)
YES	13 (1.2)	
Median (IQR)	1 (0)	
Has anyone you know well (friend, partner, family, colleague etc.) tested positive for the Coronavirus (COVID-19)?		0
NO	521 (47.8)	
YES	569 (52.2)	
Physical health and Psychological		
**(11)** In general, how would you rate your physical health as it is today? (0-10)	6.66 (SD 2.08)	0

Table S1. Sociodemographic characteristics of the sample (cont.)

	Total (N=1090)<br>n (%)/ Mean (SD)	Missing values<br>n (%)
**12)** In general, to what extent do you feel happy these days? (0-10)	5.53 (2.29)	1 (0.1)
Please imagine a ladder, with steps numbered 0 at the bottom and 10 at the top. The top represents the best possible life for you, and the bottom represents the worst possible life for you. On which step of the ladder would you say you personally feel you stand at this time?	5.82 (SD 2.06)	1 (0.1)
As a person, I am always optimistic for my future	6.89 (2.28)	1 (0.1)
Overall, I expect more good things to happen to me than bad	7.05 (2.24)	1 (0.1)
I have high self-esteem	6.54 (2.25)	1 (0.1)

Source: Authors

Note: The mean and standard deviation (SD) are included in the cells for numerical variables. Also, median and interquartile range (IQR) is provided in the cases of non-normality. For categorical variables, the number of cases and percentages.

Table S2. Explanatory factors for compliance with social distance measures. Hierarchical Multiple Regression Models

During the days of the coronavirus (COVID-19) pandemic, I have been …	1) … Staying at home as much as practically possible				2) … Visiting friends, family, or colleagues outside my home				3) … Keeping the number of grocery store visits at an absolute minimum				4) … Keeping physical distance from all other people outside my home				5) … Avoiding handshaking with people outside my home				6) … Avoiding kissing and hugging with people outside my home			
	Beta	p	95% CI Lower	95% CI Higher	Beta	p	95% CI Lower	95% CI Higher	Beta	p	95% CI Lower	95% CI Higher	Beta	p	95% CI Lower	95% CI Higher	Beta	p	95% CI Lower	95% CI Higher	Beta	p	95% CI Lower	95% CI Higher
**(Constant)**		0.153	-0.08	0.50		0.000	-1.20	-0.37		0.533	-0.21	0.41		0.000	-1.09	-0.32		0.000	-1.21	-0.39		0.024	-0.84	-0.06
**1) Sociodemographic**																								
Female	0.191	0.000	0.21	0.42	0.067	0.082	-0.02	0.28	0.005	0.884	-0.10	0.12	0.100	0.005	0.06	0.34	0.014	0.719	-0.12	0.18	0.073	0.043	0.00	0.29
Age	-0.066	0.181	-0.13	0.03	-0.034	0.566	-0.15	0.08	0.289	0.000	0.18	0.35	-0.003	0.954	-0.11	0.10	0.015	0.799	-0.10	0.13	-0.044	0.429	-0.15	0.06
Marital status (Reference=Married) Single, without a partner	-0.009	0.813	-0.15	0.12	-0.148	0.001	-0.53	-0.14	-0.109	0.002	-0.38	-0.08	0.090	0.025	0.03	0.39	0.047	0.280	-0.09	0.30	0.034	0.405	-0.11	0.26
Single in a relationship	-0.179	0.000	-0.46	-0.20	-0.218	0.000	-0.68	-0.31	-0.009	0.783	-0.16	0.12	-0.034	0.382	-0.25	0.09	-0.004	0.916	-0.19	0.17	-0.030	0.446	-0.24	0.11
Number of children	-0.020	0.593	-0.08	0.04	-0.109	0.013	-0.19	-0.02	-0.031	0.379	-0.09	0.04	-0.060	0.134	-0.14	0.02	-0.040	0.348	-0.12	0.04	-0.104	0.012	-0.18	-0.02
Level of studies (Reference=Posgraduate) Primary	-0.038	0.266	-0.46	0.13	0.047	0.235	-0.17	0.68	0.007	0.821	-0.28	0.35	0.113	0.002	0.22	1.00	0.111	0.005	0.19	1.02	0.064	0.086	-0.05	0.75
High school	0.047	0.266	-0.06	0.22	0.030	0.535	-0.14	0.27	0.079	0.049	0.00	0.30	0.194	0.000	0.22	0.59	0.120	0.013	0.05	0.45	0.067	0.145	-0.05	0.33
Vocational training	-0.032	0.439	-0.20	0.09	-0.010	0.834	-0.23	0.18	0.020	0.605	-0.11	0.19	0.215	0.000	0.28	0.66	0.152	0.001	0.13	0.53	0.112	0.013	0.05	0.44
University (Degree, Bachelor)	-0.010	0.757	-0.17	0.12	0.001	0.982	-0.21	0.21	0.043	0.168	-0.05	0.27	0.043	0.226	-0.07	0.31	0.134	0.000	0.16	0.57	0.071	0.052	0.00	0.39
Work situation (Reference=Others) Employed full time	-0.045	0.513	-0.29	0.15	0.325	0.000	0.33	0.97	0.027	0.676	-0.19	0.29	0.037	0.615	-0.22	0.37	0.197	0.013	0.08	0.71	0.040	0.604	-0.22	0.38
Employed part-time	-0.061	0.140	-0.46	0.06	-0.001	0.982	-0.38	0.37	-0.046	0.232	-0.46	0.11	0.015	0.729	-0.29	0.41	-0.068	0.151	-0.64	0.10	-0.039	0.388	-0.51	0.20
Unemployed / Looking for work	-0.076	0.115	-0.46	0.05	0.263	0.000	0.51	1.25	0.048	0.292	-0.13	0.42	0.036	0.486	-0.22	0.46	0.090	0.103	-0.06	0.67	-0.040	0.450	-0.48	0.21
Student	-0.085	0.101	-0.53	0.05	0.219	0.000	0.36	1.20	0.085	0.081	-0.03	0.59	0.049	0.372	-0.21	0.56	0.163	0.006	0.17	0.99	-0.028	0.626	-0.49	0.30
Retired	-0.009	0.881	-0.25	0.22	0.262	0.000	0.34	1.02	-0.306	0.000	-0.99	-0.48	0.158	0.010	0.10	0.73	0.162	0.014	0.09	0.76	0.082	0.196	-0.11	0.54
Where would you place yourself on this ladder to represent where you think you stand at this time in your life, compared to other people in Spain? (10-At the top, 0-At the bottom	0.062	0.046	0.00	0.10	0.074	0.040	0.00	0.15	0.024	0.407	-0.03	0.08	-0.109	0.001	-0.17	-0.04	-0.096	0.007	-0.17	-0.03	-0.097	0.004	-0.16	-0.03
Habitat: Urban	-0.054	0.069	-0.24	0.01	0.090	0.009	0.06	0.41	-0.107	0.000	-0.39	-0.12	-0.159	0.000	-0.58	-0.25	-0.111	0.001	-0.46	-0.12	-0.107	0.001	-0.45	-0.11

Table S2. Explanatory factors for compliance with social distance measures. Hierarchical Multiple Regression Models (cont.)

**During the days of the coronavirus (COVID-19) pandemic, I have been …**	**1) … Staying at home as much as practically possible**				**2) … Visiting friends, family, or colleagues outside my home**				**3) … Keeping the number of grocery store visits at an absolute minimum**				**4) … Keeping physical distance from all other people outside my home**				**5) … Avoiding handshaking with people outside my home**				**6) … Avoiding kissing and hugging with people outside my home**			
	**Beta**	**p**	**95% CI Lower**	**95% CI Higher**	**Beta**	**p**	**95% CI Lower**	**95% CI Higher**	**Beta**	**p**	**95% CI Lower**	**95% CI Higher**	**Beta**	**p**	**95% CI Lower**	**95% CI Higher**	**Beta**	**p**	**95% CI Lower**	**95% CI Higher**	**Beta**	**p**	**95% CI Lower**	**95% CI Higher**
**Physical hygiene and support for political measures regarding the coronavirus**																								
**2)** Average based personal hygiene index (0-10)	0.170	0.000	0.09	0.19	0.045	0.202	-0.02	0.11	0.243	0.000	0.17	0.28	0.306	0.000	0.24	0.37	0.279	0.000	0.21	0.35	0.256	0.000	0.19	0.32
**3)** Coronavirus policy support index based on Mean (0-10)	0.095	0.010	0.02	0.14	-0.007	0.868	-0.09	0.08	-0.014	0.685	-0.08	0.05	0.192	0.000	0.11	0.27	0.035	0.415	-0.05	0.12	0.061	0.132	-0.02	0.14
**4)** Interaction Personal hygiene * Policy support regarding coronavirus (0-100)	-0.389	0.000	-0.40	-0.30	-0.101	0.004	-0.17	-0.03	-0.109	0.000	-0.15	-0.05	-0.129	0.000	-0.19	-0.07	-0.141	0.000	-0.21	-0.07	-0.239	0.000	-0.30	-0.17
**Conspiracy theories**																								
**5)** Specific COVID-19 conspiracy theories																								
The coronavirus (COVID-19) is a bioweapon engineered by scientists (0-10)	0.128	0.009	0.03	0.18	-0.093	0.102	-0.20	0.02	-0.065	0.161	-0.14	0.02	-0.091	0.081	-0.19	0.01	-0.051	0.360	-0.16	0.06	-0.123	0.022	-0.23	-0.02
The coronavirus (COVID-19) is a conspiracy to take away citizen's rights for good and establish an authoritarian government (0-10)	-0.076	0.150	-0.15	0.02	0.028	0.654	-0.09	0.15	-0.320	0.000	-0.39	-0.20	-0.294	0.000	-0.41	-0.18	-0.230	0.000	-0.35	-0.11	-0.233	0.000	-0.35	-0.12
The coronavirus (COVID-19) is a hoax invented by interest groups for financial gains (0-10)	-0.026	0.579	-0.09	0.05	0.149	0.006	0.04	0.26	0.148	0.001	0.06	0.22	0.073	0.145	-0.03	0.17	0.030	0.577	-0.08	0.14	-0.031	0.550	-0.13	0.07
The coronavirus (COVID-19) was created as a cover up for the impending global economic crash (0-10)	0.041	0.463	-0.05	0.12	-0.037	0.563	-0.16	0.09	0.210	0.000	0.10	0.29	0.153	0.010	0.04	0.27	0.131	0.040	0.01	0.26	0.199	0.001	0.08	0.32

Table S2. Explanatory factors for compliance with social distance measures. Hierarchical Multiple Regression Models (cont.)

During the days of the coronavirus (COVID-19) pandemic, I have been …	1) … Staying at home as much as practically possible				2) … Visiting friends, family, or colleagues outside my home				3) … Keeping the number of grocery store visits at an absolute minimum				4) … Keeping physical distance from all other people outside my home				5) … Avoiding handshaking with people outside my home				6) … Avoiding kissing and hugging with people outside my home			
	Beta	p	95% CI Lower	95% CI Higher	Beta	p	95% CI Lower	95% CI Higher	Beta	p	95% CI Lower	95% CI Higher	Beta	p	95% CI Lower	95% CI Higher	Beta	p	95% CI Lower	95% CI Higher	Beta	p	95% CI Lower	95% CI Higher
**6)** Beliefs in general conspiracy theories (CMQ). I think… 1-Certainly not 0%, 11-Certain 100%)																								
… many very important things happen in the world, which the public is never informed about	0.248	0.000	0.13	0.27	0.051	0.296	-0.04	0.15	0.213	0.000	0.13	0.27	-0.040	0.368	-0.13	0.05	0.051	0.284	-0.04	0.14	0.013	0.774	-0.08	0.10
… politicians usually do not tell us the true motives for their decisions.	-0.083	0.027	-0.13	-0.01	0.057	0.197	-0.03	0.14	-0.126	0.000	-0.18	-0.05	0.052	0.198	-0.03	0.13	0.039	0.372	-0.05	0.12	0.051	0.218	-0.03	0.13
… government agencies closely monitor all citizens	0.082	0.030	0.01	0.13	-0.037	0.400	-0.12	0.05	0.144	0.000	0.07	0.20	-0.013	0.742	-0.09	0.07	-0.109	0.012	-0.19	-0.02	-0.091	0.029	-0.17	-0.01
… events which superficially seem to lack a connection are often the result of secret activities.	-0.041	0.413	-0.11	0.05	-0.011	0.856	-0.13	0.10	-0.046	0.331	-0.13	0.04	0.201	0.000	0.10	0.31	0.123	0.033	0.01	0.24	0.075	0.176	-0.03	0.18
… there are secret organizations that greatly influence political decisions.	-0.127	0.007	-0.18	-0.03	-0.104	0.059	-0.21	0.00	-0.125	0.005	-0.20	-0.03	-0.157	0.002	-0.26	-0.06	-0.086	0.110	-0.19	0.02	-0.052	0.319	-0.15	0.05
**Mass media and social networks used to get informed**																								
**7)** Summation Index of the frequency of use of 3 modern means to get informed (online digital newspapers, blogs, social networks as Facebook, Twitter, Instagram, etc.) (3-18)	0.092	0.002	0.03	0.12	0.103	0.003	0.03	0.17	-0.025	0.369	-0.07	0.03	0.038	0.231	-0.02	0.10	0.088	0.010	0.02	0.15	0.035	0.287	-0.03	0.10
Summation Index of the frequency of use of 4 traditional means to get informed (television, paper newspapers, magazines, radio) (4-24)	-0.131	0.000	-0.16	-0.06	-0.018	0.625	-0.09	0.05	-0.068	0.024	-0.12	-0.01	-0.094	0.006	-0.16	-0.03	0.010	0.790	-0.06	0.08	-0.089	0.011	-0.16	-0.02

Table S2. Explanatory factors for compliance with social distance measures. Hierarchical Multiple Regression Models (cont.)

During the days of the coronavirus (COVID-19) pandemic, I have been …	1) … Staying at home as much as practically possible				2) … Visiting friends, family, or colleagues outside my home				3) … Keeping the number of grocery store visits at an absolute minimum				4) … Keeping physical distance from all other people outside my home				5) … Avoiding handshaking with people outside my home				6) … Avoiding kissing and hugging with people outside my home			
	Beta	p	95% CI Lower	95% CI Higher	Beta	p	95% CI Lower	95% CI Higher	Beta	p	95% CI Lower	95% CI Higher	Beta	p	95% CI Lower	95% CI Higher	Beta	p	95% CI Lower	95% CI Higher	Beta	p	95% CI Lower	95% CI Higher
**Ideology, vote, trust in institutions, and national identification**																								
**8)** Overall, how would you describe yourself in terms of political ideology? (0-10)	-0.131	0.000	-0.16	-0.06	-0.018	0.625	-0.09	0.05	-0.068	0.024	-0.12	-0.01	-0.094	0.006	-0.16	-0.03	0.010	0.790	-0.06	0.08	-0.089	0.011	-0.16	-0.02
Vote recall (Reference=PSOE): PP	-0.214	0.000	-0.24	-0.11	0.013	0.796	-0.08	0.11	-0.355	0.000	-0.40	-0.26	-0.043	0.335	-0.13	0.04	-0.093	0.052	-0.19	0.00	0.035	0.448	-0.05	0.12
VOX	0.108	0.008	0.07	0.48	0.074	0.120	-0.06	0.53	0.074	0.053	0.00	0.44	0.182	0.000	0.31	0.85	0.211	0.000	0.38	0.96	0.137	0.002	0.16	0.71
Unidas Podemos	0.093	0.026	0.03	0.47	0.086	0.082	-0.04	0.60	0.257	0.000	0.54	1.01	0.215	0.000	0.41	1.00	0.195	0.000	0.33	0.95	0.134	0.004	0.14	0.74
Ciudadanos	-0.106	0.000	-0.47	-0.13	0.035	0.318	-0.12	0.37	-0.002	0.943	-0.19	0.18	-0.012	0.707	-0.27	0.18	0.094	0.007	0.09	0.57	0.054	0.102	-0.04	0.42
Others	0.023	0.434	-0.13	0.30	0.053	0.117	-0.06	0.55	0.068	0.013	0.06	0.53	0.051	0.103	-0.05	0.52	0.068	0.043	0.01	0.61	0.042	0.197	-0.10	0.48
Did not vote	-0.088	0.006	-0.35	-0.06	-0.069	0.065	-0.41	0.01	0.043	0.151	-0.04	0.27	0.084	0.015	0.05	0.44	0.157	0.000	0.24	0.66	0.111	0.002	0.12	0.52
Index of Trust in institutions and organizations based on the average of 16 institutions (1-4)	-0.022	0.606	-0.19	0.11	0.063	0.207	-0.08	0.35	0.138	0.001	0.12	0.44	0.239	0.000	0.32	0.71	0.214	0.000	0.25	0.67	0.203	0.000	0.24	0.64
Index of National Identification based in the mean of two-item (0-10)	-0.068	0.078	-0.12	0.01	0.052	0.248	-0.04	0.14	0.165	0.000	0.09	0.22	-0.012	0.770	-0.09	0.07	-0.005	0.910	-0.09	0.08	0.025	0.553	-0.06	0.11
**Risk perception and testing positive for COVID-19**																								
**9)** By April 30, 2021: How likely do you think it is that you will get infected by the Coronavirus (Covid-19)? (0-100)	-0.046	0.186	-0.09	0.02	-0.158	0.000	-0.24	-0.08	0.078	0.017	0.01	0.13	-0.087	0.020	-0.16	-0.01	0.029	0.462	-0.05	0.11	-0.035	0.355	-0.11	0.04
By April 30, 2021: How likely do you think it is that the average person in Spain will get infected by the Coronavirus (Covid-19)? (0-100)	-0.042	0.223	-0.09	0.02	0.041	0.317	-0.04	0.12	0.035	0.292	-0.03	0.09	0.037	0.322	-0.04	0.11	0.080	0.045	0.00	0.16	0.052	0.179	-0.02	0.13

Table S2. Explanatory factors for compliance with social distance measures. Hierarchical Multiple Regression Models (cont.)

**During the days of the coronavirus (COVID-19) pandemic, I have been …**	**1) … Staying at home as much as practically possible**				**2) … Visiting friends, family, or colleagues outside my home**				**3) … Keeping the number of grocery store visits at an absolute minimum**				**4) … Keeping physical distance from all other people outside my home**				**5) … Avoiding handshaking with people outside my home**				**6) … Avoiding kissing and hugging with people outside my home**			
	**Beta**	**p**	**95% CI Lower**	**95% CI Higher**	**Beta**	**p**	**95% CI Lower**	**95% CI Higher**	**Beta**	**p**	**95% CI Lower**	**95% CI Higher**	**Beta**	**p**	**95% CI Lower**	**95% CI Higher**	**Beta**	**p**	**95% CI Lower**	**95% CI Higher**	**Beta**	**p**	**95% CI Lower**	**95% CI Higher**
**10)** Have you tested positive for the Coronavirus (COVID-19), meaning that you (now or earlier) have had a medically confirmed case of this disease?	0.040	0.110	-0.07	0.68	0.002	0.957	-0.53	0.56	0.008	0.729	-0.33	0.48	-0.039	0.152	-0.86	0.13	-0.012	0.686	-0.64	0.42	-0.015	0.603	-0.64	0.37
Has anyone you know well (friend, partner, family, colleague etc.) tested positive for the Coronavirus (COVID-19)?	-0.018	0.532	-0.12	0.06	0.024	0.477	-0.08	0.18	-0.012	0.662	-0.12	0.08	0.111	0.000	0.10	0.34	0.007	0.835	-0.12	0.14	0.095	0.003	0.07	0.31
**Physical health and Psychological**																								
**(11)** In general, how would you rate your physical health as it is today? (0-10)	-0.024	0.442	-0.07	0.03	-0.010	0.785	-0.08	0.06	0.017	0.565	-0.04	0.07	0.015	0.655	-0.05	0.08	0.009	0.813	-0.06	0.08	0.028	0.421	-0.04	0.10
**12)** In general, to what extent do you feel happy these days? (0-10)	-0.164	0.000	-0.19	-0.08	-0.100	0.019	-0.18	-0.02	-0.103	0.003	-0.16	-0.03	-0.011	0.786	-0.09	0.07	-0.032	0.443	-0.11	0.05	-0.064	0.111	-0.14	0.01
Please imagine a ladder, with steps numbered 0 at the bottom and 10 at the top. The top represents the best possible life for you, and the bottom represents the worst possible life for you. On which step of the ladder would you say you personally feel you stand at this time?	0.121	0.003	0.03	0.16	0.001	0.985	-0.09	0.09	0.014	0.702	-0.06	0.08	-0.038	0.372	-0.12	0.05	-0.068	0.136	-0.16	0.02	-0.024	0.586	-0.11	0.06
As a person, I am always optimistic for my future	0.007	0.889	-0.07	0.09	0.155	0.009	0.04	0.27	-0.129	0.009	-0.21	-0.03	-0.010	0.851	-0.12	0.10	0.204	0.000	0.09	0.32	0.032	0.571	-0.08	0.14
Overall, I expect more good things to happen to me than bad	-0.030	0.554	-0.11	0.06	-0.025	0.681	-0.14	0.09	0.186	0.000	0.08	0.26	-0.116	0.035	-0.22	-0.01	-0.195	0.001	-0.31	-0.08	-0.095	0.094	-0.21	0.02
I have high self-esteem	0.075	0.027	0.01	0.11	0.105	0.008	0.03	0.18	0.064	0.043	0.00	0.12	0.048	0.187	-0.02	0.12	-0.021	0.589	-0.10	0.06	0.066	0.075	-0.01	0.14

Source: Authors.
